# Translational studies of exosomes in sports medicine – a mini-review

**DOI:** 10.3389/fimmu.2023.1339669

**Published:** 2024-01-08

**Authors:** Haoqiang Huang, Peng Chen, Xinting Feng, Yinhua Qian, Zhijian Peng, Ting Zhang, Qing Wang

**Affiliations:** ^1^ Department of Orthopaedics, Kunshan Hospital of Traditional Chinese Medicine, Kunshan, Jiangsu, China; ^2^ Department of Sports Medicine, Peking University Shenzhen Hospital, Shenzhen, China; ^3^ Department of Sports Medicine, Huashan Hospital, Fudan University, Shanghai, China; ^4^ Department of Integrative Medicine, Huashan Hospital, Fudan University, Shanghai, China

**Keywords:** exosomes, sports medicine, chronic diseases, injury recovery, immune regulation, translation

## Abstract

This review in sports medicine focuses on the critical role of exosomes in managing chronic conditions and enhancing athletic performance. Exosomes, small vesicles produced by various cells, are essential for cellular communication and transporting molecules like proteins and nucleic acids. Originating from the endoplasmic reticulum, they play a vital role in modulating inflammation and tissue repair. Their significance in sports medicine is increasingly recognized, particularly in healing athletic injuries, improving articular cartilage lesions, and osteoarthritic conditions by modulating cellular behavior and aiding tissue regeneration. Investigations also highlight their potential in boosting athletic performance, especially through myocytes-derived exosomes that may enhance adaptability to physical training. Emphasizing a multidisciplinary approach, this review underlines the need to thoroughly understand exosome biology, including their pathways and classifications, to fully exploit their therapeutic potential. It outlines future directions in sports medicine, focusing on personalized treatments, clinical evaluations, and embracing technological advancements. This research represents a frontier in using exosomes to improve athletes’ health and performance capabilities.

## 1 Introduction

Exosomes represent a specialized subset of extracellular vesicles, with dimensions typically ranging from 30 to 150 nanometers, that are elaborated and liberated by a broad spectrum of cells via the endoplasmic reticulum vesicle system (1–4). These nanostructures play a quintessential role in cellular communication, encapsulating and conveying an assortment of biomolecules, including proteins, lipids, RNA, and DNA (5–9). Their interaction with recipient cells significantly modulates the recipient’s cellular functions (9, 10). The ontogeny of exosomes commences within the endoplasmic reticulum vesicle system, where they originate as incipient endosomes in the cytoplasmic milieu. Subsequently, they progress to mature into multivesicular bodies (MVBs), which are repositories for myriad intraluminal vesicles that ultimately coalesce with the plasma membrane, culminating in the extracellular release of exosomes. This elaborate process is under the stringent regulation of a network of proteins and lipids (2, 4). Exosomes are taxonomically classified per their progenitor cell lineage, inherent biological functions, and distinctive surface markers, rendering them an intriguing focus of current biomedical research. Depending on the source cell, exosomes can be sub-classified into various types, such as those derived from immune cells (e.g., exosomes produced by T cells, B cells, or dendritic cells), tumor cells (exosomes from tumor cells, typically associated with promoting tumor growth and metastasis), and stem cells (exosomes from various stem cells like mesenchymal stem cells and embryonic stem cells, noted for their robust tissue repair and regenerative capabilities). Additionally, based on their biological function, exosomes can be classified as pro-inflammatory or anti-inflammatory, immunomodulatory, pro-tumorigenic, or anti-tumorigenic, and more (11–14). The analysis of exosomal surface markers, such as CD63, CD81, and CD9, which are commonly regarded as universal markers, also aids in their categorization. Understanding the diverse classifications of exosomes is pivotal in comprehending their varying biological functions and potential clinical applications. Delving into the study of different types of exosomes opens new avenues and strategies for clinical treatments, particularly in the realms of sports medicine and regenerative medicine, where exosomal research holds broad application prospects and profound scientific significance (15–17). In the field of sports medicine, exosome research is emerging as a novel and vibrant area of study. Investigating the role of exosomes in cellular communication, as well as their potential in repairing sports injuries and enhancing athletic performance, is expected to provide new theoretical foundations and experimental evidence for the clinical translation of sports medicine (7, 10, 18, 19).

Sports medicine is an interdisciplinary field of research dedicated to exploring the dynamic interplay between physical activity and human health (7, 20–26). This domain is committed to the prevention and treatment of sports injuries and the enhancement of athletic performance (7, 12, 23, 27–30). The scope of sports medicine encompasses but is not limited to, disciplines such as physiology, biomechanics, sports psychology, and sports nutrition (13, 31–35). Professionals in sports medicine frequently collaborate with physical therapists, sports psychologists, nutritionists, and experts from related fields to provide comprehensive services to athletes and the general populace. The primary objectives of sports medicine include the prevention and treatment of sports injuries, achieved through a profound understanding of the mechanisms underlying these injuries. This understanding fosters the development of novel preventive strategies and therapeutic approaches aimed at reducing the incidence of injuries and accelerating the recovery process (20, 22, 27). Additionally, sports medicine focuses on optimizing athletic performance through scientifically validated training methods, appropriate nutritional supplementation, and psychological adjustment strategies. This support empowers athletes and fitness enthusiasts to achieve peak performance levels. Furthermore, sports medicine advocates for a healthy lifestyle, utilizing education and outreach to encourage public participation in regular physical activity, thereby fostering physical and mental well-being and preventing chronic diseases (25, 26, 29, 36). As technology advances and society evolves, sports medicine will continue to expand into new domains and technologies to meet the growing demands for sports and health.

The intersection and significance of exosomes in the realm of sports medicine primarily manifest in their potential contributions to the recovery from sports injuries and the enhancement of athletic performance. Exosomes, serving as critical vectors in intercellular communication, are carriers of functional molecules such as mRNA, microRNA, and specific proteins. These molecules are instrumental in the early diagnosis and targeted treatment of various diseases. Recent studies have illuminated the significant role of exosomes in sports medicine, demonstrating that physical exercise can influence the content of exosomes, thus revealing their crucial role in this field (37).

Firstly, exosomes show remarkable potential in the realm of sports injury recovery. Research has underscored the therapeutic value of exosomes in the treatment of joint cartilage damage and osteoarthritis (OA). Joint cartilage injury, a common clinical issue, can lead to joint dysfunction, significant pain, and secondary osteoarthritis. Exosomes, originating from the endoplasmic reticulum and actively participating in cellular communication under both physiological and pathological conditions, have gained considerable attention across various domains. The significance of exosomes in the progression of osteoarthritis and their therapeutic value in cartilage repair and osteoarthritis treatment are progressively being recognized. The functional differences between various types and sources of exosomes are determined by their specific contents, influencing their role in the onset and progression of osteoarthritis and the treatment value and future therapeutic design strategies related to cartilage injuries/osteoarthritis (38). Secondly, the improvement of athletic performance through exosomes is of notable significance. Exosomes secreted by skeletal muscle cells can bind or fuse with the plasma membranes of target cells or be endocytosed, thereby transferring their effective payloads. This exosome-mediated communication between cells and organs can be viewed as a mode of transportation for myokines, potentially impacting athletic performance and the body’s adaptability to exercise (39). In summary, the intersection and importance of exosomes in sports medicine are primarily evident in their potential contributions to the recovery from sports injuries and the improvement of athletic performance. As research on exosomes continues to deepen and the field of sports medicine evolves, exosomes may emerge as a pivotal therapeutic strategy for facilitating sports injury recovery and enhancing athletic performance.

The purpose of this scholarly review is to meticulously interrogate the role and significance of exosomes within the domain of sports medicine, offering novel perspectives and foundational support for their clinical application. The specific knowledge gaps that our review seeks to address are distinct from previous studies, with a particular emphasis on translational research and personalized treatment of exosomes in sports medicine rather than merely discussing related mechanistic studies.

## 2 Biological basis of exosomes

### 2.1 Biogenesis and release of exosomes

The genesis and extracellular dispensation of exosomes are governed by a sophisticated and nuanced cascade of cellular events, necessitating the orchestrated participation of myriad organelles and biomolecular constituents. Herein, we delineate the sequential phases of this process:

The origin of exosome biogenesis is situated within the cellular endosomal framework. The odyssey begins with the invagination of the cell’s plasma membrane, leading to the genesis of late endosomes, also termed multivesicular bodies (MVBs). Within the confines of these MVBs, a secondary invagination transpires, culminating in the creation of intraluminal vesicles (ILVs). These nascent vesicles harbor an array of biomolecular entities, including proteins, lipids, and RNA moieties, derived from both the cytosol and the cell’s exterior membrane. Subsequently, MVBs are trafficked to the periphery of the cell, coalescing with the plasma membrane and facilitating the liberation of ILVs into the extracellular milieu, at which juncture they assume the designation of exosomes (40).

The incipient phase of exosomal biogenesis is catalyzed by the cell’s endosomal recycling apparatus. This process commences with the selective internalization of molecular constituents from the plasma membrane into the incipient endosomes. These early endosomes undergo a series of biochemical and biophysical processes to transform into late endosomes and subsequently into MVBs. The formation of ILVs within MVBs is accomplished through the inward budding of the cell membrane. MVBs are then transported to the cell membrane, where they fuse and release exosomes into the extracellular space (40, 41).

The release mechanisms of exosomes may be regulated by the Endosomal Sorting Complex Required for Transport (ESCRT) machinery, although there is some contention regarding this. During exosome biogenesis, several key proteins such as Alix, flotillin, and TSG101 have been identified as participants in the process. These proteins are likely intricately involved with the fusion of the cell membrane and the release of exosomes (42).

Exosome biogenesis and release involve interactions among multiple organelles and biomolecules, as well as a variety of biochemical and biophysical processes. The cargo of exosomes, including proteins and miRNAs, and their sorting and packaging, are integral components of the exosome biogenesis process. With those functions, exosomes can efficiently transport specific biomolecules to target cells and act as a key role in intercellular communication (43).

### 2.2 Contents of exosomes

The molecular cargo of exosomes mirrors their progenitor cellular milieu, encompassing a panoply of moleculcar constituents such as lipids, proteins, and nucleic acid sequences. The exosomal membrane is characterized by a lipid bilayer architecture, enriched with cholesterol, sphingomyelin, ceramide, and diglycerides (44). Exosomes are notably replete with an array of transmembrane proteins, including tetraspanins, antigen-presenting complexes, an assortment of glycoproteins, and molecules facilitating cellular adhesion; alongside a cadre of luminal proteins comprising heat shock proteins, elements of the cytoskeleton, components of the endosomal sorting complexes required for transport (ESCRT) machinery, membranous transporters, fusogenic proteins, and an array of growth factors and cytokines (44, 45). Beyond these proteins, exosomal cargo encompasses nucleic acids, inclusive of DNA, messenger RNA, and microRNA.

The repertoire of exosomal contents further extends to lipids, metabolic intermediates, as well as proteins intrinsic to the cytoplasm and cellular interface. These molecular entities are capable of assimilation by recipient cells, exerting functional modulation (41, 42). Despite the obscurity surrounding the physiological raison of exosomes, burgeoning studies delineate their quintessential role as conveyors in intercellular signaling, orchestrating the communicative network among disparate cell types (41, 43). Proteomic scrutiny of exosomes secreted across various cellular origins has elucidated a conserved set of proteins, thereby postulating exosomes as a bona fide secretory subcellular organelle, while also identifying unique protein signatures indicative of the discrete functional capacities engendered by exosomes from divergent cellular provenances.

### 2.3 Biological activity and function of exosomes

Exosomes are extracellular vesicles produced by all cells, responsible for intercellular communication. Carrying genetic information and proteins, they transport molecules from one cell to another via vesicular transport, influencing biological processes such as immune responses, cell proliferation, and neural signaling (46). The bioactive cargo of exosomes may provide prognostic information for a range of diseases, including chronic inflammation, cardiovascular and renal diseases, neurodegenerative disorders, lipid metabolism diseases, and tumors (42). They contain components secreted by their parent cells (e.g., proteins, DNA, and RNA) and can be taken up by distant cells, affecting cellular functions and behavior (46).

Exosomes are recognized as a ubiquitous intracorporeal conveyance mechanism, replete with multifunctionality, ferrying an array of nucleic acids, proteins, lipids, and metabolic byproducts. They fulfill a critical function as conduits for both proximal and distal intercellular discourse in both physiological and pathological states (46). The myriad functions and biological activities of exosomes are pivotal in the realms of cellular and pathobiological sciences, especially in the domains of intercellular signaling, inflammatory mediation, and the pathogenesis of disease.

Through the transport and transference of diverse bioactive moieties such as growth factors, cytokines, and microRNAs, exosomes exert regulatory control over the functional dynamics and behavioral responses of target cells (12, 47). For example, they can orchestrate immune responses by conveying molecules with immunomodulatory efficacy, such as antigen-presenting complexes and immunosuppressive agents, thereby modulating the functional status of immune cells. In addition, exosomes can impinge upon cellular proliferation, motility, and phenotypic differentiation by transmitting growth factors and cytokines (48). Within the neural milieu, exosomes demonstrate substantial bioactivity, influencing neuronal viability and functionality through the delivery of neurotrophic factors and neurotransmitter-related molecules (49). Notably, exosomes are implicated in oncogenesis and tumorigenesis, shaping the biological characteristics of neoplastic cells and the architectonics of the tumor microenvironment by the translocation of oncogenic and invasive factors (50).

Many types of exosomes are utilized as well in sports medicine, such as bone marrow MSC exosomes, adipose stem cell exosomes, embryonic MSC exosomes, umbilical cord MSC exosomes, dental pulp stem cell exosomes, and so on. In this article, we will describe the relevant studies that have been reported.

## 3 Exosomes in sports medicine-related research

### 3.1 Role of exosomes in tissue repair and regeneration

#### 3.1.1 Muscle injury

The reparative role of exosomes in myotrauma has garnered considerable scrutiny, with findings affirming their ability to instigate muscular tissue regeneration through multifarious mechanisms. Predominantly, exosomes expedite myotrauma remediation and restoration by stimulating myogenic proliferation, catalyzing the phenotypic maturation of tendinous cells, fostering neurite outgrowth, and facilitating the proliferation of Schwannian cells (51). Exosomes derived from platelet-enriched plasma and mesenchymal stromal cells have been observed to significantly expedite the recuperation of muscular functionality (51).

Exosomes exert their influence by attenuating cellular pyroptosis and ameliorating ischemic myopathy. Empirical evidence suggests that exosomes sourced from mesenchymal stromal cells (MSCs) harbor the therapeutic potential for myopathic injuries, endorsing myoblastic differentiation in patients with Duchenne Muscular Dystrophy and in murine models of MDX (52). Furthermore, exosomes emanating from C2C12 myoblasts have been implicated in the promotion of myofibrillar regeneration, expediting lipogenesis within injured myocytes, mitigating myofibrosis, and accelerating reparative processes, which are attributed to the exosomal mediation of satellite cell proliferation and fibro-adipogenic progenitor cell differentiation (53).

Exosomes isolated from human adipose-derived mesenchymal stromal cells (AD-MSCs) have demonstrated promising therapeutic implications for myogenic regeneration. These exosomes are postulated as efficacious modalities for regenerative therapy, potentially inaugurating novel avenues for myotrauma remediation (54). Additionally, exosomes from bone marrow stromal cells (BMSCs) have been documented to enhance muscular healing by promoting M2 macrophagic polarization, whereas pro-inflammatory C2C12-derived exosomes have been associated with M1 macrophagic polarization and the suppression of myogenic repair mechanisms (12, 27, 55).

#### 3.1.2 Frozen shoulder

Adhesive Capsulitis (AC), commonly manifested as Shoulder Stiffness (SS), is a pervasive affliction characterized by aggravated pain and a diminished range of articular motion (56). Pathologically, AC is classified as an inflammatory and fibrotic disorder. Investigations have unveiled that exosomes from Bone Marrow Stromal Cells (BMSCs) can suppress the expression of TGFBR1 through the mediation of let-7a-5p, consequently impeding the progression of AC (14, 28). Exosomes have emerged as a significant therapeutic entity in the management of diverse fibrotic maladies, with exosomes from various sources and their molecular cargoes—such as miRNAs, lncRNAs, and proteins—being contemplated as targeted therapeutic interventions. These entities can influence an array of cellular types and signal transduction pathways implicated in fibrosis (57, 58).

#### 3.1.3 Tendon injury

Exosomes hold significant potential in the treatment of tendon injuries, encompassing Achilles and rotator cuff injuries. In the realm of tendon injury therapy, exosomes function through multiple mechanisms. These primarily include the suppression of inflammatory responses, modulation of macrophage polarization, regulation of gene expression, remodeling of the cellular microenvironment, restructuring of the extracellular matrix, and promotion of angiogenesis (59).

In the context of rotator cuff injury repair, exosomes exhibit considerable therapeutic potential. Studies have found that Mesenchymal Stem Cells (MSCs) can enhance healing post-rotator cuff repair through the release of exosomes (60). Additionally, purified exosome products are being explored to improve the surgical outcomes of rotator cuff tendon-bone healing and to reduce postoperative re-tear rates. This is achieved by focusing on biological and biomechanical factors (61).

In the treatment of Achilles tendon injuries, exosomes have also demonstrated the ability to promote the healing of injured tendons. Specifically, exosomes from tendon stem cells have been found to facilitate tendon injury healing through mechanisms that balance the synthesis and degradation of the tendon extracellular matrix (62). Concurrently, given that poor outcomes in many soft tissue injuries (such as Achilles tendon ruptures, rotator cuff tears, and flexor tendon injuries) are attributed to macrophage-induced inflammation, researchers are investigating exosome-based therapies to suppress inflammation and thereby improve the treatment outcomes of tendon injuries (10).

#### 3.1.4 Tendon-bone healing

Exosomes have demonstrated significant therapeutic potential in tendon-bone healing, particularly in the healing process following Anterior Cruciate Ligament Reconstruction (ACLR). Research indicates that exosomes derived from Bone Marrow Stromal Cells (BMSCs) can facilitate tendon-bone healing by modulating the polarization of M1/M2 type macrophages, a mechanism that has been validated in rat models of ACLR (63). Typically, ACLR may fail due to the inability to regenerate normal tissue at the tendon-bone junction and the formation of fibrous scar tissue at this interface (64). However, the combination of BMSC-derived exosomes with cartilage fragments has been shown to enhance healing at the tendon-bone interface, thereby increasing the success rate of ACLR (65).

The role of exosomes in facilitating tendon-bone healing primarily encompasses (1) inhibition of inflammatory responses and regulation of macrophage polarization, (2) control of gene expression, remodeling of the cellular microenvironment, and restructuring of the extracellular matrix, and (3) promotion of angiogenesis (59). Although studies have observed the effects of BMSC-derived exosomes (BMSC-Exos) on tendon-bone healing post-ACLR in rats, both *in vivo* and *in vitro*, elucidating the possible mechanisms, it remains unclear whether BMSC-Exos can facilitate tendon-bone healing in humans post-ACLR. Additionally, some studies have explored the effects of exosomes on tendon-bone healing and osteogenesis at the tendon-bone junction using rat ACLR models. For instance, by locally injecting IONP-Exos, BMSC-Exos, or PBS into the surgical knee joint, the retention of exosomes at the surgical site was observed. It was found that exosomes can promote bone formation at the tendon-bone junction (66).

#### 3.1.5 Arthritis

Exosomes have demonstrated potential value in the treatment of arthritis, primarily manifesting in alleviating cartilage damage, inhibiting bone overgrowth, and modulating immune responses. Certain types of exosomes show potential advantages in reducing inflammation and regulating immune responses, which could be significant for the treatment of disease models including Rheumatoid Arthritis (RA). Exosomes can inhibit the proliferation of T lymphocytes indicative of inflammation and induce other anti-inflammatory effects (67, 68). Additionally, exosomes are involved in numerous physiological and pathological processes, influencing the development of various diseases, including Osteoarthritis (OA), by regulating intercellular communication (69).

In the treatment of Rheumatoid Arthritis, exosomes can act as therapeutic carriers. These extracellular vesicles from mice cells affect biological mechanisms and signal transduction by transporting genetic and proteomic components (67). Exosomes also play a role in RA-related arthritis, where these specialized function extracellular vesicles are responsible for transporting autoantigens and mediators to distant cells (68).

#### 3.1.6 Cartilage injury

Firstly, exosomes can promote cartilage repair and regeneration by regulating cell migration, proliferation, differentiation, and extracellular matrix synthesis. For example, exosomes from Mesenchymal Stem Cells (MSCs) can modulate immune responses, reduce cell apoptosis, enhance cell proliferation, and initiate the proliferation and migration of chondroprogenitor cells (19, 38). Additionally, exosomes can alleviate cartilage injury, reduce bone overgrowth, inhibit the production of M1 macrophages, and promote the generation of M2 macrophages, while also decreasing levels of pro-inflammatory cytokines such as IL-1β, IL-6, and TNF-α, and increasing levels of the anti-inflammatory cytokine IL-10 (70).

In the treatment of rheumatoid arthritis, exosomes can be used as therapeutic carriers; they are extracellular vesicles in mice that influence biological mechanisms and signaling and can play a role by transporting genetic and protein components (67). Exosome also plays a role in rheumatoid arthritis, these specialized functioning extracellular vesicles are responsible for transporting self-antigens and mediators to distant cells (68).

### 3.2 Potential of exosomes in enhancing athletic performance

#### 3.2.1 Metabolic regulation

Exosomes, small vesicles originating from the endoplasmic reticulum, circulate through blood and other bodily fluids, providing a unique platform for intercellular communication. Recent research highlights the pivotal role of exosomes in metabolic regulation during physical activity, especially in endurance exercises (39, 71, 72). Here is an overview of the role of exosomes in exercise-induced metabolic regulation.

Bioactive Molecules in Exosomes: During exercise, the bioactive molecules within exosomes, such as peptides and nucleic acids (collectively termed exerkines), undergo alterations. Studies indicate that endurance exercise induces the release of exosomes, particularly peptides and nucleic acids from skeletal muscle and other tissues (73, 74). These bioactive molecules can exert endocrine-like effects, impacting the pathophysiology of conditions like obesity and Type 2 Diabetes (73–75).

Intercellular Communication Role of Exosomes: Functioning as endocrine-like vesicles, exosomes can carry proteins, microRNAs, and other nucleic acids, facilitating communication between cells and tissues, and even among organs. This contributes to the formation of a coordinated metabolic network within the body (72, 74, 76).

Future Therapeutic Potential of Exosomes: Researchers hypothesize that future therapies for obesity and Type 2 Diabetes might involve the use of modified exosomes enriched with exerkines. These exosomes, through their contained bioactive molecules, could positively regulate metabolic health, offering new therapeutic possibilities for these metabolic diseases (73, 74, 76).

Release of Exosomes and Exercise Intensity: Interestingly, studies have also discovered that with increasing exercise intensity, the concentration of exosomes in circulation correspondingly rises. This further suggests a critical role of exosomes in metabolic regulation during exercise (73, 77).

#### 3.2.2 Anti-fatigue and antioxidant potential of exosomes

Recent research has unveiled the significance of exosomes in combating fatigue and oxidative stress (78–81). In the realm of anti-fatigue, exosomes are believed to improve cellular energy metabolism and enhance cells’ resistance to fatigue and damage. The mechanistic repertoire of exosomes in cellular bioenergetics encompasses the enhancement of mitochondrial efficacy, the amplification of adenosine triphosphate (ATP) synthesis, and the optimization of oxidative phosphorylation efficiency (78, 79). Additionally, the exosomal content of select RNA species and proteins may actuate specific metabolic cascades, thereby underpinning recuperative processes subsequent to physical exertion (78, 80, 81).

In the context of antioxidative activity, exosomes possess proficiency in the sequestration and neutralization of reactive oxygen species (80–82). This antioxidative mechanism is predominantly ascribed to the presence of enzymatic constituents within exosomes, such as catalases, sulfiredoxins, and an array of redox-modulating molecules. These enzymes are adept at obliterating surplus free radicals, thereby mitigating oxidative stress and attenuating cellular damage. Moreover, exosomes exert a modulatory effect on intracellular antioxidant pathways, including the Nrf2 axis, thereby reinforcing the cellular defense against oxidative insults (82). The exosomal complement of bioactive RNA and proteins also fine-tunes the redox equilibrium, which bolsters their antioxidative properties.

Empirical investigations have corroborated that exosomal antioxidants, for instance, glutathione and superoxide dismutase, are capable of neutralizing excessive reactive species, ameliorating oxidative detriment (82, 83). This plays an instrumental role in diminishing muscular fatigue post-exercise and decelerating cellular senescence. Furthermore, exosomal antioxidants are pivotal in sustaining intracellular redox homeostasis, thus endorsing normative cellular operations (84, 85). Additionally, exosomes harbor an ensemble of anti-fatigue molecular entities such as heat shock proteins and antioxidative enzymes, orchestrating the cellular stress response and fostering recuperation (82, 86). In response to fatigue-inducing stimuli, cellular systems can escalate the release of these anti-fatigue proteins via exosomes, thereby enhancing endurance and resilience.

#### 3.2.3 Enhancing athlete performance

Exosomes are implicated as pivotal entities in the mediation of myocyte repair, a process of particular pertinence to athletes undergoing rigorous training regimens (12, 52, 53, 87). In the aftermath of high-intensity exercise, athletes frequently endure microtraumas within muscular tissues, precipitating inflammation and consequent discomfort. Investigations have substantiated that exosomes possess the capability to encapsulate and convey growth factors, microRNAs, and an array of other bioactive compounds to myocytes experiencing trauma. This facilitates a cascade of cellular activities inclusive of proliferation, motility, and morphological specialization, thereby expediting the restoration and rejuvenation of compromised tissues. In addition, exosomes have been observed to potentiate athletic stamina. The myriad bioactive molecules harbored within exosomes are known to initiate a spectrum of metabolic processes that amplify the efficacy of mitochondrial oxidative phosphorylation (39, 88, 89). This suggests that in the milieu of sustained and intensive exertion, the myocytes of athletes are equipped to uphold an elevated rate of adenosine triphosphate (ATP) synthesis, thereby augmenting endurance.

**Figure 1:**
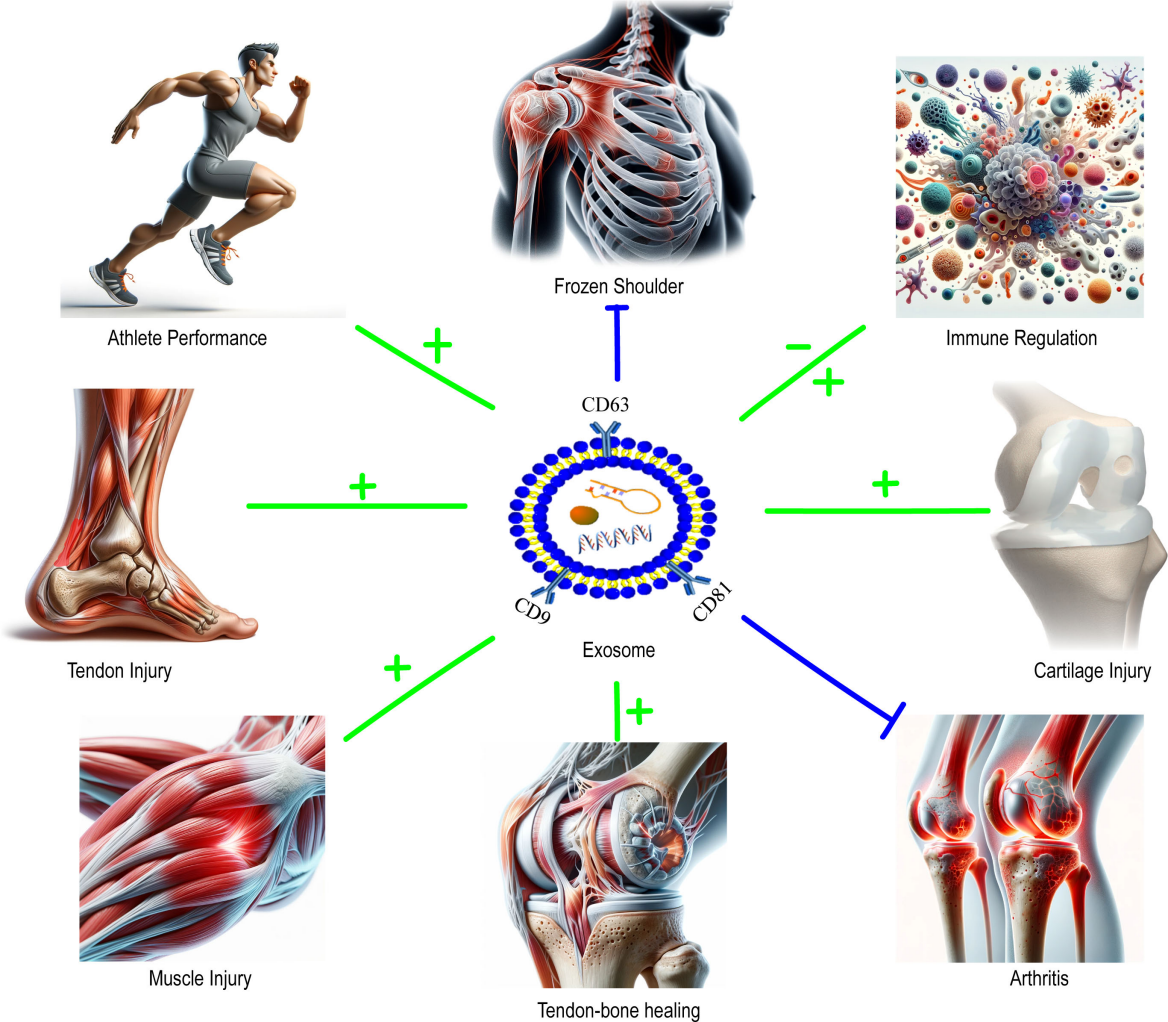
The role of exosomes on sports medicine-related issues.

In addition, exosomes can augment athletes’ antioxidative capabilities. During exercise, increased oxygen consumption leads to the production of reactive oxygen species (ROS), which can cause cellular damage (8, 48, 82, 83). However, antioxidative enzymes and other molecules within exosomes can effectively scavenge these free radicals, protecting cells from damage and expediting the recovery process. Lastly, exosomes can modulate immune responses, reducing post-exercise inflammatory reactions. Specific proteins and RNA molecules within exosomes can influence the activation and secretion of immune cells, thereby inhibiting the production and release of inflammatory cytokines and reducing post-exercise inflammation (**Figure 1**) (50, 90, 91).

## 4 Perspectives and challenges

### 4.1 Technological innovation and optimization

Future research endeavors will continually drive innovation and optimization in exosome extraction, purification, and preparation techniques. This includes developing more efficient extraction methods, enhancing purification efficacy, and reducing production costs. Technological advancements will contribute to improving the quality and yield of exosome formulations, thereby increasing their feasibility for clinical applications (3, 92–94). Emerging technologies shaping exosome research include advanced nanoengineering approaches for precision therapeutics. Techniques like aptamer-guided targeting allow for the development of exosomal delivery systems that are more specific and effective. Additionally, microfluidic engineering and post-isolation modifications of exosomes are enhancing their application in nanomedicine. These innovations are crucial in refining exosome-based therapies, making them more targeted and efficient for use in sports medicine and beyond.

### 4.2 Multidisciplinary collaboration

Exosome research necessitates collaboration across multiple disciplines, including cell biology, molecular biology, clinical medicine, and engineering. Such collaboration will aid in a deeper understanding of the biological properties, mechanisms, and clinical applications of exosomes and help address various challenges in exosome therapy. Cross-disciplinary teamwork will be a significant trend in future exosome research (95, 96). For example, bioengineering and nanoengineering techniques can be used to develop targeted exosomal delivery systems. These systems can be engineered with aptamers or chemical antibodies for precision medicine, enhancing the specificity and efficacy of exosome-based therapies (97–99).

### 4.3 Personalized therapy

The future development of exosome therapy will increasingly focus on personalized treatment strategies. Tailoring exosome formulations and treatment plans based on patient’s genetic, molecular, and physiological characteristics can enhance therapeutic outcomes and minimize unnecessary side effects (100–102). The extent to which AI and machine learning can aid in the progression of personalized therapy needs to be explored further.

### 4.4 Clinical trials and regulatory approval

More clinical trials are needed in the future to validate the safety and efficacy of exosome therapy. Strict regulatory approval and compliance are essential to ensure patient safety and treatment reliability. Formulating clear clinical trial protocols and regulatory policies will be a crucial task moving forward (103, 104).

### 4.5 Long-term safety and efficacy monitoring

Monitoring the long-term safety and efficacy of exosome therapy poses one of the future challenges. As patients undergo prolonged exosome therapy, effective monitoring methods need to be established to assess long-term impacts and side effects. This will require large-scale patient follow-up studies and data analysis (105, 106).

### 4.6 Standardization of production and storage

To meet future clinical demands, standardized processes for the production and storage of exosome formulations are necessary. This includes ensuring consistency, stability, and purity of exosome formulations to meet diverse patient needs. Standardized processes will aid in enhancing the scalability and feasibility of exosome therapy (71, 107, 108).

### 4.7 Limitation and clinic-lab gap

In the field of exosome research in sports medicine, the translation of laboratory findings to clinical practice faces several challenges. These include ensuring the stability and consistent quality of exosome preparations, understanding the complex mechanisms of exosome-cell interactions, and addressing safety concerns such as immune responses and potential long-term effects. Regulatory hurdles also play a significant role, as there is a need for standardized protocols and guidelines for exosome therapy. Moreover, ethical considerations, particularly in the context of enhancing athletic performance, must be thoroughly addressed. These challenges require multidisciplinary collaboration and advancements in both research methodologies and regulatory frameworks.

In summary, exosomes hold vast potential for clinical treatment in sports medicine-related diseases. However, future development must address challenges in technological innovation, multidisciplinary collaboration, personalized therapy, clinical trials and regulatory approval, long-term monitoring, and standardization of production. By overcoming these challenges, exosome therapy has the potential to offer more effective and safer treatment strategies for diseases in the field of sports medicine, improving patients’ quality of life. This will require close collaboration among the scientific community, medical institutions, and governmental regulatory bodies to propel the future development of exosome therapy.

## 5 Summary

Research advancements in the field of sports medicine reveal the substantial potential of exosomes, which have already achieved some encouraging results in clinical translation. Here’s a summary of the research progress and clinical translation potential of exosomes in sports medicine, emphasizing future research directions.

### 5.1 Research progress

Anti-inflammatory and Antioxidative Effects: Exosomes, rich in various anti-inflammatory factors and antioxidants, emerge as powerful tools for treating exercise-related diseases. Accompanying sports injuries are inflammation and oxidative stress, where exosomes can facilitate healing and recovery by inhibiting inflammatory responses and reducing oxidative damage.

Tissue Repair: Growth factors and signaling molecules in exosomes have the potential to promote tissue repair and regeneration. This is particularly vital for the treatment of sports-related muscle, skeletal injuries, and cartilage damage. Exosomes can activate stem cells and aid in repairing damaged tissues.

Optimization of Athletic Performance: Some studies have also explored the application of exosomes in enhancing athletic performance. Growth factors and proteins within exosomes can stimulate muscle growth and repair, improving muscle strength and endurance, and thereby aiding in enhancing athletic performance.

### 5.2 Clinical translation potential

Treating Sports Injuries: Exosome therapy can be employed in treating sports-related injuries such as muscle strains, fracture healing, and cartilage repair. It can accelerate the healing process, and reduce pain and inflammation, thereby shortening recovery time.

Joint Health: For joint diseases like osteoarthritis, exosome therapy shows potential in anti-inflammatory and joint-protective actions. Injecting exosomes into damaged joints can alleviate pain and improve joint functionality.

Cardiovascular Rehabilitation: Antioxidants and cardioprotective factors in exosomes aid in cardiovascular rehabilitation. They can reduce cardiac damage, improve cardiac function, and lower the risk of cardiovascular diseases.

In summary, the research progress and clinical translational potential of exosomes in sports medicine are exciting, but further in-depth studies and clinical validation are still needed. Future research directions should focus on an in-depth understanding of the mechanism, individualized treatment, more clinical trials, and the establishment of standardized preparation and quality control processes to fully explore the application prospects of exosomes in the field of sports medicine.

## Author contributions

HH: Writing – original draft, Writing – review & editing. PC: Writing – original draft, Writing – review & editing. XF: Investigation, Writing – original draft. YQ: Data curation, Investigation, Writing – original draft. ZP: Conceptualization, Funding acquisition, Writing – original draft, Writing – review & editing. TZ: Conceptualization, Data curation, Funding acquisition, Supervision, Writing – original draft. QW: Conceptualization, Data curation, Formal Analysis, Funding acquisition, Methodology, Project administration, Resources, Supervision, Validation, Visualization, Writing – original draft, Writing – review & editing.

## References

[1] Estébanez B Jiménez-Pavón D Huang C Cuevas MJ González-Gallego J. Effects of exercise on exosome release and cargo in *in vivo* and ex vivo models: A systematic review. J Cell Physiol (2021) 236:3336–53. doi: 10.1002/jcp.30094 33037627

[2] Pegtel DM Gould SJ. Exosomes. Annu Rev Biochem (2019) 88:487–514. doi: 10.1146/annurev-biochem-013118-111902 31220978

[3] Cong M Tan S Li S Gao L Huang L Zhang H-G. Technology insight: Plant-derived vesicles-How far from the clinical biotherapeutics and therapeutic drug carriers? Adv Drug Delivery Rev (2022) 182:114108. doi: 10.1016/j.addr.2021.114108 34990792

[4] Wang J Li X Wang S Cui J Ren X Su J. Bone-targeted exosomes: strategies and applications. Adv Heal Mater (2023) 12(18):e2203361. doi: 10.1002/adhm.202203361 36881547

[5] Liu Y Weng W Gao R Liu Y. New insights for cellular and molecular mechanisms of aging and aging-related diseases: herbal medicine as potential therapeutic approach. Oxid Med Cell Longev (2019) 2019:4598167. doi: 10.1155/2019/4598167 31915506 PMC6930799

[6] Wu Y Hu C Wang Y-B. Recent advances in the application of biomimetic nanomedicines in disease treatment. BioMed Eng Commun (2022) 1:4. doi: 10.53388/bmec2022004

[7] Chen Y Luo Z Sun Y Zhou Y Han Z Yang X. The effect of denture-wearing on physical activity is associated with cognitive impairment in the elderly: A cross-sectional study based on the CHARLS database. Front Neurosci (2022) 16:925398. doi: 10.3389/fnins.2022.925398 36051648 PMC9425833

[8] Wang Y Zhao R Liu D Deng W Xu G Liu W. Exosomes derived from miR-214-enriched bone marrow-derived mesenchymal stem cells regulate oxidative damage in cardiac stem cells by targeting caMKII. Oxid Med Cell Longev (2018) 2018:4971261. doi: 10.1155/2018/4971261 30159114 PMC6109555

[9] Ying W Riopel M Bandyopadhyay G Dong Y Birmingham A Seo JB. Adipose tissue macrophage-derived exosomal miRNAs can modulate *in vivo* and *in vitro* insulin sensitivity. Cell (2017) 171:372–384.e12. doi: 10.1016/j.cell.2017.08.035 28942920

[10] Iyer SR Scheiber AL Yarowsky P Henn RF Otsuru S Lovering RM. Exosomes isolated from platelet-rich plasma and mesenchymal stem cells promote recovery of function after muscle injury. Am J Sports Med (2020) 48:2277–86. doi: 10.1177/0363546520926462 32543878

[11] Chen Y Sun Y Luo Z Lin J Qi B Ying C. Potential mechanism Q4 underlying exercise upregulated circulating blood exosome miR-215-5p to prevent necroptosis of neuronal cells and a model for early diagnosis of alzheimer’s disease. Front Aging Neurosci (2022) 14, 1–15. doi: 10.3389/fnagi.2022.860364 PMC9126031 35615585

[12] Luo Z Qi B Sun Y Chen Y Lin J Qin H. Engineering bioactive M2 macrophage-polarized, anti-inflammatory, miRNA-based liposomes for functional muscle repair: from exosomal mechanisms to biomaterials. Small (2022) 18:2201957. doi: 10.1002/smll.202201957 35802903

[13] Feng X Peng Z Yuan L Jin M Hu H Peng X. Research progress of exosomes in pathogenesis, diagnosis, and treatment of ocular diseases. Front Bioeng Biotechnol (2023) 11:1100310. doi: 10.3389/fbioe.2023.1100310 36761297 PMC9902372

[14] Luo Z Sun Y Qi B Lin J Chen Y Xu Y. Human bone marrow mesenchymal stem cell-derived extracellular vesicles inhibit shoulder stiffness via let-7a/Tgfbr1 axis. Bioact Mater (2022) 17:344–59. doi: 10.1016/j.bioactmat.2022.01.016 PMC8965035 35386460

[15] Forterre A Jalabert A Berger E Baudet M Chikh K Errazuriz E. Proteomic analysis of C2C12 myoblast and myotube exosome-like vesicles: A new paradigm for myoblast-myotube cross talk? PloS One (2014) 9:e84153. doi: 10.1371/journal.pone.0084153 24392111 PMC3879278

[16] Puno MR Weick EM Das M Lima CD. SnapShot: the RNA exosome. Cell (2019) 179:282–282.e1. doi: 10.1016/j.cell.2019.09.005 31539497

[17] Pomatto M Gai C Negro F Cedrino M Grange C Ceccotti E. Differential therapeutic effect of extracellular vesicles derived by bone marrow and adipose mesenchymal stem cells on wound healing of diabetic ulcers and correlation to their cargoes. Int J Mol Sci (2021) 22:1–26. doi: 10.3390/ijms22083851 PMC8068154 33917759

[18] Wang C Hu Q Song W Yu W He Y. Adipose stem cell–derived exosomes decrease fatty infiltration and enhance rotator cuff healing in a rabbit model of chronic tears. Am J Sports Med (2020) 48:1456–64. doi: 10.1177/0363546520908847 32272021

[19] Cai J Xu J Ye Z Wang L Zheng T Zhang T. Exosomes derived from kartogenin-preconditioned mesenchymal stem cells promote cartilage formation and collagen maturation for enthesis regeneration in a rat model of chronic rotator cuff tear. Am J Sports Med (2023) 51:1267–76. doi: 10.1177/03635465231155927 36917828

[20] Sun Y Lin J Luo Z Chen J. Preoperative lymphocyte to monocyte ratio can be a prognostic factor in arthroscopic repair of small to large rotator cuff tears. Am J Sports Med (2020) 48:3042–50. doi: 10.1177/0363546520953427 32931300

[21] Lin J Yang X Liu S Luo Z Chen Q Sun Y. Long non-coding RNA MFAT1 promotes skeletal muscle fibrosis by modulating the miR-135a-5p-Tgfbr2/Smad4 axis as a ceRNA. J Cell Mol Med (2021) 25:4420–33. doi: 10.1111/jcmm.16508 PMC8093971 33837645

[22] Luo Z Lin J Sun Y Zhu K Wang C Chen J. Outcome comparison of latissimus dorsi transfer and pectoralis major transfer for irreparable subscapularis tendon tear: A systematic review. Am J Sports Med (2022) 50:2032–41. doi: 10.1177/03635465211018216 34138660

[23] Wang C Sun Y Ding Z Lin J Luo Z Chen J. Influence of femoral version on the outcomes of hip arthroscopic surgery for femoroacetabular impingement or labral tears: A systematic review and meta-analysis. Orthop J Sport Med (2021) 9:232596712110091. doi: 10.1177/23259671211009192 PMC8202282 34179203

[24] Luo Z Zhang T Chen S. Exercise prescription: pioneering the “Third pole” for clinical health management. Research (2023) 6:1–4. doi: 10.34133/research.0284 PMC10684289 38034085

[25] Luo Z Wan R Liu S Feng X Peng Z Wang Q. Mechanisms of exercise in the treatment of lung cancer – a mini-review. Front Immunol (2023) 14:1244764. doi: 10.3389/fimmu.2023.1244764 37691942 PMC10483406

[26] Luo Z Sun Y-Y Xia W Xu J-Y Xie D-J Jiao C-M. Physical exercise reverses immuno-cold tumor microenvironment via inhibiting SQLE in non-small cell lung cancer. Mil Med Res (2023) 10:39. doi: 10.1186/s40779-023-00474-8 37592367 PMC10436398

[27] Luo Z Lin J Sun Y Wang C Chen J. Bone marrow stromal cell-derived exosomes promote muscle healing following contusion through macrophage polarization. Stem Cells Dev (2021) 30:135–48. doi: 10.1089/scd.2020.0167 33323007

[28] Sun Y Luo Z Chen Y Lin J Zhang Y Qi B. si-Tgfbr1-loading liposomes inhibit shoulder capsule fibrosis via mimicking the protective function of exosomes from patients with adhesive capsulitis. Biomater Res (2022) 26:39. doi: 10.1186/s40824-022-00286-2 35986376 PMC9389696

[29] Luo Z He Z Qin H Chen Y Qi B Lin J. Exercise-induced IL-15 acted as a positive prognostic implication and tumor-suppressed role in pan-cancer. Front Pharmacol (2022) 13:1053137. doi: 10.3389/fphar.2022.1053137 36467072 PMC9712805

[30] Wan R Zhang H Liu S Jiao C Chen H Luo Z. Role of fibro-adipogenic progenitors in skeletal muscle aging. Aging Pathobiol Ther (2023) 5:72–8. doi: 10.31491/apt.2023.06.116

[31] Qin H Luo Z Sun Y He Z Qi B Chen Y. Low-intensity pulsed ultrasound promotes skeletal muscle regeneration via modulating the inflammatory immune microenvironment. Int J Biol Sci (2023) 19:1123–45. doi: 10.7150/ijbs.79685 PMC10008697 36923940

[32] Qin H Du L Luo Z He Z Wang Q Chen S. The therapeutic effects of low-intensity pulsed ultrasound in musculoskeletal soft tissue injuries: Focusing on the molecular mechanism. Front Bioeng Biotechnol (2022) 10:1080430. doi: 10.3389/fbioe.2022.1080430 36588943 PMC9800839

[33] Lin J Luo Z Liu S Chen Q Liu S Chen J. Long non-coding RNA H19 promotes myoblast fibrogenesis via regulating the miR-20a-5p-Tgfbr2 axis. Clin Exp Pharmacol Physiol (2021) 48:921–31. doi: 10.1111/1440-1681.13489 33615521

[34] Hu T Luo Z Li K Wang S Wu D. Zanthoxylum nitidum extract attenuates BMP-2-induced inflammation and hyperpermeability. Biosci Rep (2020) 40(10):BSR20201098. doi: 10.1042/BSR20201098 33030503 PMC7584816

[35] Chen Y Sun Y Xu Y Lin WW Luo Z Han Z. Single-cell integration analysis of heterotopic ossification and fibrocartilage developmental lineage: endoplasmic reticulum stress effector xbp1 transcriptionally regulates the notch signaling pathway to mediate fibrocartilage differentiation. Oxid Med Cell Longev (2021) 2021:1–29. doi: 10.1155/2021/7663366 PMC8563124 34737845

[36] Chen P Wang D Shen H Yu L Gao Q Mao L. Physical activity and health in Chinese children and adolescents: expert consensus statement (2020). Br J Sports Med (2020) 54:1321–31. doi: 10.1136/bjsports-2020-102261 PMC7606574 32471813

[37] Li Y Han C Wang J Zhou J Liang C Ranganna K. Exosomes mediate the beneficial effects of exercise. Adv Exp Med Biol (2017) 1000:333–53. doi: 10.1007/978-981-10-4304-8_18 29098629

[38] Zhou Q Cai Y Jiang Y Lin X. Exosomes in osteoarthritis and cartilage injury: advanced development and potential therapeutic strategies. Int J Biol Sci (2020) 16:1811–20. doi: 10.7150/ijbs.41637 PMC7211167 32398951

[39] Aoi W Tanimura Y. Roles of skeletal muscle-derived exosomes in organ metabolic and immunological communication. Front Endocrinol (Lausanne) (2021) 12:697204. doi: 10.3389/fendo.2021.697204 34594301 PMC8476901

[40] Krylova SV Feng D. The machinery of exosomes: biogenesis, release, and uptake. Int J Mol Sci (2023) 24:1337. doi: 10.3390/ijms24021337 36674857 PMC9865891

[41] Hessvik NP Llorente A. Current knowledge on exosome biogenesis and release. Cell Mol Life Sci (2018) 75:193–208. doi: 10.1007/s00018-017-2595-9 28733901 PMC5756260

[42] Zhang Y Liu Y Liu H Tang WH. Exosomes: biogenesis, biologic function and clinical potential. Cell Biosci (2019) 9:19. doi: 10.1186/s13578-019-0282-2 30815248 PMC6377728

[43] Xie S Zhang Q Jiang L. Current knowledge on exosome biogenesis, cargo-sorting mechanism and therapeutic implications. Membranes (Basel) (2022) 12:498. doi: 10.3390/membranes12050498 35629824 PMC9144303

[44] Isola AL Chen S. Exosomes: the messengers of health and disease. Curr Neuropharmacol (2017) 15:157–65. doi: 10.2174/1570159x14666160825160421 PMC5327461 27568544

[45] Fazaeli H Kalhor N Naserpour L Davoodi F Sheykhhasan M Hosseini SKE. A comparative study on the effect of exosomes secreted by mesenchymal stem cells derived from adipose and bone marrow tissues in the treatment of osteoarthritis-induced mouse model. BioMed Res Int (2021) 2021:9688138. doi: 10.1155/2021/9688138 PMC8490078 34616850

[46] Kalluri R LeBleu VS. The biology, function, and biomedical applications of exosomes. Science (2020) 367:eaau6977. doi: 10.1126/science.aau6977 32029601 PMC7717626

[47] Mori MA Ludwig RG Garcia-Martin R Brandão BB Kahn CR. Extracellular miRNAs: from biomarkers to mediators of physiology and disease. Cell Metab (2019) 30:656–73. doi: 10.1016/j.cmet.2019.07.011 PMC6774861 31447320

[48] Yamaguchi T Izumi Y Nakamura Y Yamazaki T Shiota M Sano S. Repeated remote ischemic conditioning attenuates left ventricular remodeling via exosome-mediated intercellular communication on chronic heart failure after myocardial infarction. Int J Cardiol (2015) 178:239–46. doi: 10.1016/j.ijcard.2014.10.144 25464262

[49] Ghamloush F Ghayad SE Rammal G Fahs A Ayoub AJ Merabi Z. The PAX3-FOXO1 oncogene alters exosome miRNA content and leads to paracrine effects mediated by exosomal miR-486. Sci Rep (2019) 9:1–12. doi: 10.1038/s41598-019-50592-4 31578374 PMC6775163

[50] Nabet BY Qiu Y Shabason JE Wu TJ Yoon T Kim BC. Exosome RNA unshielding couples stromal activation to pattern recognition receptor signaling in cancer. Cell (2017) 170:352–366.e13. doi: 10.1016/j.cell.2017.06.031 28709002 PMC6611169

[51] Wan R Hussain A Behfar A Moran SL Zhao C. The therapeutic potential of exosomes in soft tissue repair and regeneration. Int J Mol Sci (2022) 23:3869. doi: 10.3390/ijms23073869 35409228 PMC8998690

[52] Wang Z Yang J Sun X Sun X Yang G Shi X. Exosome-mediated regulatory mechanisms in skeletal muscle: a narrative review. J Zhejiang Univ Sci B (2023) 24:1–14. doi: 10.1631/jzus.B2200243 36632747 PMC9837378

[53] Ji S Ma P Cao X Wang J Yu X Luo X. Myoblast-derived exosomes promote the repair and regeneration of injured skeletal muscle in mice. FEBS Open Bio (2022) 12:2213–26. doi: 10.1002/2211-5463.13504 PMC9714366 36325691

[54] Byun S-E Sim C Chung Y Kim HK Park S Kim DK. Skeletal muscle regeneration by the exosomes of adipose tissue-derived mesenchymal stem cells. Curr Issues Mol Biol (2021) 43:1473–88. doi: 10.3390/cimb43030104 PMC8929094 34698065

[55] Luo Z-W Sun Y-Y Lin J-R Qi B-J Chen J-W. Exosomes derived from inflammatory myoblasts promote M1 polarization and break the balance of myoblast proliferation/differentiation. World J Stem Cells (2021) 13:1762–82. doi: 10.4252/wjsc.v13.i11.1762 PMC8641021 34909122

[56] Itoi E Arce G Bain GI Diercks RL Guttmann D Imhoff AB. Shoulder stiffness: current concepts and concerns. Arthrosc J Arthrosc Relat Surg Off Publ Arthrosc Assoc North Am Int Arthrosc Assoc (2016) 32:1402–14. doi: 10.1016/j.arthro.2016.03.024 27180923

[57] Liu Y Zheng Y Yang Y Liu K Wu J Gao P. Exosomes in liver fibrosis: The role of modulating hepatic stellate cells and immune cells, and prospects for clinical applications. Front Immunol (2023) 14:1133297. doi: 10.3389/fimmu.2023.1133297 37020547 PMC10067730

[58] Qin X-J Zhang J-X Wang R-L. Exosomes as mediators and biomarkers in fibrosis. biomark Med (2020) 14:697–712. doi: 10.2217/bmm-2019-0368 32643390

[59] Zou M Wang J Shao Z. Therapeutic potential of exosomes in tendon and tendon-bone healing: A systematic review of preclinical studies. J Funct Biomater (2023) 14:299. doi: 10.3390/jfb14060299 37367263 PMC10299056

[60] Connor DE Paulus JA Dabestani PJ Thankam FK Dilisio MF Gross RM. Therapeutic potential of exosomes in rotator cuff tendon healing. J Bone Miner Metab (2019) 37:759–67. doi: 10.1007/s00774-019-01013-z PMC6830879 31154535

[61] Ren Y Zhang S Wang Y Jacobson DS Reisdorf RL Kuroiwa T. Effects of purified exosome product on rotator cuff tendon-bone healing *in vitro* and *in vivo* . Biomaterials (2021) 276:121019. doi: 10.1016/j.biomaterials.2021.121019 34325337 PMC9707649

[62] Wang Y He G Guo Y Tang H Shi Y Bian X. Exosomes from tendon stem cells promote injury tendon healing through balancing synthesis and degradation of the tendon extracellular matrix. J Cell Mol Med (2019) 23:5475–85. doi: 10.1111/jcmm.14430 PMC6653097 31148334

[63] Li Z Li Q Tong K Zhu J Wang H Chen B. BMSC-derived exosomes promote tendon-bone healing after anterior cruciate ligament reconstruction by regulating M1/M2 macrophage polarization in rats. Stem Cell Res Ther (2022) 13:295. doi: 10.1186/s13287-022-02975-0 35841008 PMC9284827

[64] Sun Y Chen W Hao Y Gu X Liu X Cai J. Stem cell–conditioned medium promotes graft remodeling of midsubstance and intratunnel incorporation after anterior cruciate ligament reconstruction in a rat model. Am J Sports Med (2019) 47:2327–37. doi: 10.1177/0363546519859324 31306585

[65] Zhang C Jiang C Jin J Lei P Cai Y Wang Y. Cartilage fragments combined with BMSCs-Derived exosomes can promote tendon-bone healing after ACL reconstruction. Mater Today Bio (2023) 23:100819. doi: 10.1016/j.mtbio.2023.100819 PMC10550801 37810754

[66] Wu X-D Kang L Tian J Wu Y Huang Y Liu J. Exosomes derived from magnetically actuated bone mesenchymal stem cells promote tendon-bone healing through the miR-21-5p/SMAD7 pathway. Mater Today Bio (2022) 15:100319. doi: 10.1016/j.mtbio.2022.100319 PMC9218580 35757032

[67] Singh A Behl T Sehgal A Singh S Sharma N Naqwi M. Exploring the role of exosomes in rheumatoid arthritis. Inflammopharmacology (2023) 31:119–28. doi: 10.1007/s10787-022-01100-0 36414831

[68] Heydari R Koohi F Rasouli M Rezaei K Abbasgholinejad E Bekeschus S. Exosomes as rheumatoid arthritis diagnostic biomarkers and therapeutic agents. Vaccines (2023) 11:687. doi: 10.3390/vaccines11030687 36992270 PMC10057381

[69] Ni Z Zhou S Li S Kuang L Chen H Luo X. Exosomes: roles and therapeutic potential in osteoarthritis. Bone Res (2020) 8:25. doi: 10.1038/s41413-020-0100-9 32596023 PMC7305215

[70] Wang R Xu B. TGF-β1-modified MSC-derived exosomal miR-135b attenuates cartilage injury via promoting M2 synovial macrophage polarization by targeting MAPK6. Cell Tissue Res (2021) 384:113–27. doi: 10.1007/s00441-020-03319-1 33404840

[71] Villatoro AJ Alcoholado C Martín-Astorga MC Fernández V Cifuentes M Becerra J. Comparative analysis and characterization of soluble factors and exosomes from cultured adipose tissue and bone marrow mesenchymal stem cells in canine species. Vet Immunol Immunopathol (2019) 208:6–15. doi: 10.1016/j.vetimm.2018.12.003 30712794

[72] Castaño C Mirasierra M Vallejo M Novials A Párrizas M. Delivery of muscle-derived exosomal miRNAs induced by HIIT improves insulin sensitivity through down-regulation of hepatic FoxO1 in mice. Proc Natl Acad Sci (2020) 117:30335–43. doi: 10.1073/pnas.2016112117 PMC7720135 33199621

[73] Safdar A Saleem A Tarnopolsky MA. The potential of endurance exercise-derived exosomes to treat metabolic diseases. Nat Rev Endocrinol (2016) 12:504–17. doi: 10.1038/nrendo.2016.76 27230949

[74] Safdar A Tarnopolsky MA. Exosomes as mediators of the systemic adaptations to endurance exercise. Cold Spring Harb Perspect Med (2018) 8:a029827. doi: 10.1101/cshperspect.a029827 28490541 PMC5830902

[75] Song M Han L Chen F Wang D Wang F Zhang L. Adipocyte-derived exosomes carrying sonic hedgehog mediate M1 macrophage polarization-induced insulin resistance via ptch and PI3K pathways. Cell Physiol Biochem (2018) 48:1416–32. doi: 10.1159/000492252 30064125

[76] Isaac R Reis FCG Ying W Olefsky JM. Exosomes as mediators of intercellular crosstalk in metabolism. Cell Metab (2021) 33:1744–62. doi: 10.1016/j.cmet.2021.08.006 PMC8428804 34496230

[77] Ma C Wang J Liu H Chen Y Ma X Chen S. Moderate exercise enhances endothelial progenitor cell exosomes release and function. Med Sci Sport Exerc (2018) 50:2024–32. doi: 10.1249/MSS.0000000000001672 30222687

[78] Huang Q Wu M Wu X Zhang Y Xia Y. Muscle-to-tumor crosstalk: The effect of exercise-induced myokine on cancer progression. Biochim Biophys Acta - Rev Cancer (2022) 1877:188761. doi: 10.1016/j.bbcan.2022.188761 35850277

[79] Mytidou C Koutsoulidou A Zachariou M Prokopi M Kapnisis K Spyrou GM. Age-Related Exosomal and Endogenous Expression Patterns of miR-1, miR-133a, miR-133b, and miR-206 in Skeletal Muscles. Front Physiol (2021) 12:708278. doi: 10.3389/fphys.2021.708278 34867435 PMC8637414

[80] Fulzele S Mendhe B Khayrullin A Johnson M Kaiser H Liu Y. Extracellular vesicles secreted from mouse muscle cells suppress osteoclast formation: Roles of mitochondrial energy metabolism. Bone (2017) 20:1–3. doi: 10.1016/j.stem.2016.12.009 32092478

[81] Fulzele S Mendhe B Khayrullin A Johnson M Kaiser H Liu Y. Muscle-derived miR-34a increases with age in circulating extracellular vesicles and induces senescence of bone marrow stem cells. Aging (Albany NY) (2019) 11:1791–803. doi: 10.18632/aging.101874 PMC6461183 30910993

[82] Wang T Jian Z Baskys A Yang J Li J Guo H. MSC-derived exosomes protect against oxidative stress-induced skin injury via adaptive regulation of the NRF2 defense system. Biomaterials (2020) 257:120264. doi: 10.1016/j.biomaterials.2020.120264 32791387

[83] Fahs A Ramadan F Ghamloush F Ayoub AJ Ahmad FA Kobeissy F. Effects of the oncoprotein PAX3-FOXO1 on modulation of exosomes function and protein content: Implications on oxidative stress protection and enhanced plasticity. Front Oncol (2020) 10:1784. doi: 10.3389/fonc.2020.01784 33117671 PMC7560303

[84] Mytidou C Koutsoulidou A Katsioloudi A Prokopi M Kapnisis K Michailidou K. Muscle-derived exosomes encapsulate myomiRs and are involved in local skeletal muscle tissue communication. FASEB J (2021) 35:1–21. doi: 10.1096/fj.201902468RR PMC12315491 33484211

[85] Lu J Yang X He C Chen Y Li C Li S. Rejuvenation of tendon stem/progenitor cells for functional tendon regeneration through platelet-derived exosomes loaded with recombinant Yap1. Acta Biomater (2023) 161:80–99. doi: 10.1016/j.actbio.2023.02.018 36804538

[86] Guescini M Maggio S Ceccaroli P Battistelli M Annibalini G Piccoli G. Extracellular vesicles released by oxidatively injured or intact C2C12 myotubes promote distinct responses converging toward myogenesis. Int J Mol Sci (2017) 18:2488. doi: 10.3390/ijms18112488 29165341 PMC5713454

[87] Yan B Zhang Y Liang C Liu B Ding F Wang Y. Stem cell-derived exosomes prevent pyroptosis and repair ischemic muscle injury through a novel exosome/circHIPK3/ FOXO3a pathway. Theranostics (2020) 10:6728–42. doi: 10.7150/thno.42259 PMC7295049 32550900

[88] Goulielmaki E Ioannidou A Tsekrekou M Stratigi K Poutakidou IK Gkirtzimanaki K. Tissue-infiltrating macrophages mediate an exosome-based metabolic reprogramming upon DNA damage. Nat Commun (2020) 11:42. doi: 10.1038/s41467-019-13894-9 31896748 PMC6940362

[89] Miao C Zhang W Feng L Gu X Shen Q Lu S. Cancer-derived exosome miRNAs induce skeletal muscle wasting by Bcl-2-mediated apoptosis in colon cancer cachexia. Mol Ther - Nucleic Acids (2021) 24:923–38. doi: 10.1016/j.omtn.2021.04.015 PMC8141664 34094711

[90] Conceição M Forcina L Wiklander OPB Gupta D Nordin JZ Vrellaku B. Engineered extracellular vesicle decoy receptor-mediated modulation of the IL6 trans-signalling pathway in muscle. Biomaterials (2021) 266:120435. doi: 10.1016/j.biomaterials.2020.120435 33049461

[91] Wang H Wang B Zhang A Hassounah F Seow Y Wood M. Exosome-mediated miR-29 transfer reduces muscle atrophy and kidney fibrosis in mice. Mol Ther (2019) 27:571–83. doi: 10.1016/j.ymthe.2019.01.008 PMC6403486 30711446

[92] Wu S-T Chen L Kang W-W Liu Q Gao M-T Li D-S. Recent developments of fluorescence imaging technology in the second near-infrared window. BioMed Eng Commun (2022) 1:3. doi: 10.53388/bmec2022003

[93] Zhuang Y Fu Y Huang S Gong S. The application of intelligent sensors in medical research: a review. BioMed Eng Commun (2023) 2:17. doi: 10.53388/BMEC2023017.Executive

[94] Wu F-C Ju G-Q Zhou L-H Yuan F-W. CRISPR system contributes to deciphering the pharmaceutical compounds from TCM. BioMed Eng Commun (2023) 2:8. doi: 10.53388/bmec2023008

[95] Hu X Li F Zeng J Zhou Z Wang Z Chen J. Noninvasive low-frequency pulsed focused ultrasound therapy for rheumatoid arthritis in mice. Research (2022) 2022:0013. doi: 10.34133/research.0013 PMC11407525 39290964

[96] Barreto C Jandus A. Role of natural products in combating cancer. Cancer Insight (2022) 1:35–52. doi: 10.58567/ci01010003

[97] Yue J Chen Z-S Xu X-X Li S. Functions and therapeutic potentials of exosomes in osteosarcoma. Acta Mater Med (2022) 1:552–62. doi: 10.15212/amm-2022-0024 PMC9879305 36710945

[98] Wei T Thakur SS Liu M Wen J. Oral delivery of glutathione: antioxidant function, barriers and strategies. Acta Mater Med (2022) 1:177–92. doi: 10.15212/amm-2022-0005

[99] Gao Z Zheng S Kamei K Tian C. Recent progress in cancer therapy based on the combination of ferroptosis with photodynamic therapy. Acta Mater Med (2022) 1:411–26. doi: 10.15212/amm-2022-0025

[100] Dong N Lv H Liu C Zhang P. Research progress in 3D-printed medicinal tablets. Acta Mater Med (2022) 1:154–63. doi: 10.15212/amm-2021-0010

[101] Xie X Hu L Xue H Xiong Y Panayi AC Lin Z. Prognosis and treatment of complications associated with COVID-19: a systematic review and meta-analysis. Acta Mater Med (2022) 1:124–37. doi: 10.15212/amm-2022-0002

[102] Li J Chen J Bai H Wang H Hao S Ding Y. An overview of organs-on-chips based on deep learning. Research (2022) 2022:9869518. doi: 10.34133/2022/9869518 35136860 PMC8795883

[103] Zhao T Yang Z Yu J Lu J Li L Xu X. Comparing long-term outcomes of entecavir and tenofovir disoproxil fumarate in liver transplant patients. Adv Gut Microbiome Res (2022) 2022:1–9. doi: 10.1155/2022/4779960

[104] Xie H Zhang J Gu Q Yu Q Xia L Yao S. Cohort profile: A prospective study of gut microbiota in patients with acute ischemic stroke. Adv Gut Microbiome Res (2023) 2023:1–9. doi: 10.1155/2023/3944457

[105] Cook MJ O’Brien TJ Berkovic SF Murphy M Morokoff A Fabinyi G. Prediction of seizure likelihood with a long-term, implanted seizure advisory system in patients with drug-resistant epilepsy: A first-in-man study. Lancet Neurol (2013) 12:563–71. doi: 10.1016/S1474-4422(13)70075-9 23642342

[106] Netzer K Balmith M Thabile B. An appraisal of the regulatory policies governing the use of herbal traditional medicine. Tradit Med Res (2021) 6:57. doi: 10.53388/tmr20211013249

[107] Chen C Yao X Xu Y Zhang Q Wang H Zhao L. Dahuang Zhechong Pill suppresses colorectal cancer liver metastasis via ameliorating exosomal CCL2 primed pre-metastatic niche. J Ethnopharmacol (2019) 238:111878. doi: 10.1016/j.jep.2019.111878 30986521

[108] Sengupta V Sengupta S Lazo A Woods P Nolan A Bremer N. Exosomes derived from bone marrow mesenchymal stem cells as treatment for severe COVID-19. Stem Cells Dev (2020) 29:747–54. doi: 10.1089/scd.2020.0080 PMC7310206 32380908

